# Micropillar Topography Regulates Morphology and Melanogenesis in Melanoma Cells

**DOI:** 10.3390/jfb17060269

**Published:** 2026-06-01

**Authors:** Heonuk Jeong, Koji Tsutsumi, Shohei Matsunobu, Shun-ichi Fukushima, Hui-Hsing Hung, Tomoki Matsuda

**Affiliations:** 1 Department of Biosciences, Kitasato University School of Science, 1-15-1 Kitazato, Minami-ku, Sagamihara 252-0373, Kanagawa, Japan; k.tutumi@kitasato-u.ac.jp (K.T.); hung.huihsing@kitasato-u.ac.jp (H.-H.H.); 2 Bioimaging Research Center, Kitasato University School of Medicine, 1-15-1 Kitazato, Minami-ku, Sagamihara 252-0373, Kanagawa, Japan

**Keywords:** melanoma cell, surface topography, micropatterned substrate, melanogenesis, mechanotransduction

## Abstract

Microscale physical cues at the cell–extracellular matrix adhesion interface are increasingly being recognized as important regulators of cellular behavior. B16-F10 melanoma-derived cells retain melanogenic activity, including microphthalmia-associated transcription factor (MITF) expression and inducible melanin production, and are widely used for studies of melanogenesis and pigmentation-associated cellular responses. Melanocytic cells are sensitive to the physical characteristics of the surrounding microenvironment, including adhesion-dependent mechanical cues. However, the mechanism by which physical cues derived from the adhesion interface regulate melanoma cell function remains incompletely understood. In this study, we investigated the mechanism by which defined micropatterned substrates modulate melanoma cell morphology, migration, nuclear architecture, and melanogenic activity. Polydimethylsiloxane substrates with pillar- and hole-shaped microstructures (5, 10, and 50 µm diameters and spacings; 10 µm height or depth) were fabricated and coated with fibronectin. B16-F10 melanoma cells cultured on narrow pillar patterns (5 and 10 µm) exhibited restricted cell spreading, shortened protrusions, suppressed migration, and pronounced nuclear deformation compared with flat substrates. These mechanical constraints were accompanied by significant reductions in melanin production and downregulation of melanogenesis-related genes (Mitf, Tyr, and Tyrp1). Comparable trends were observed for Matrigel-coated substrates, indicating that microscale topography exerted consistent effects on B16-F10 melanoma cell responses across the tested extracellular matrix conditions. Collectively, our results demonstrate that surface topography with narrow pillar microstructures is associated with topography-dependent changes in cell behavior and melanogenic activity, providing insights into how microscale topographic confinement influences melanoma cell morphology and melanogenic activity.

## 1. Introduction

Melanocytes are neural crest-derived pigment cells that reside in the basal layer of the epidermis, where they synthesize and transfer melanin to the surrounding keratinocytes, providing critical photoprotection against ultraviolet radiation-induced DNA damage [1,2,3]. Melanoma, which arises from the malignant transformation of melanocytes, retains melanocytic biology, including melanin production and expression of microphthalmia-associated transcription factor (MITF), while acquiring additional properties such as enhanced invasiveness and phenotypic plasticity [4,5,6,7]. In the skin, both melanocytes and melanoma cells interact with neighboring keratinocytes and the basement membrane through cell–cell and cell–extracellular matrix (ECM) adhesion. These adhesive interactions play essential roles in maintaining epidermal architecture and regulating cell morphology, survival, migration, and melanogenic activity [8,9,10,11]. Accumulating studies indicate that melanocytic cells are sensitive to the physical characteristics of the surrounding environment, including ECM stiffness and adhesion-dependent signaling, and that such physical cues can modulate cellular phenotypic state and melanogenic activity [5,11,12,13,14].

Recent advances in micro- and nano-fabrication technologies have enabled the systematic investigation of how defined topographic features influence cellular behavior [15,16,17]. Engineered substrates with controlled microstructures have been shown to modulate cell spreading, cytoskeletal organization, and focal adhesion formation, thereby regulating mechanotransduction pathways and cellular functions in multiple cell types [18,19,20,21,22,23,24]. However, despite the known mechanosensitivity of melanocytic cells, their specific responses to defined surface topographies remain poorly understood compared to those of other skin-resident cells, such as dermal fibroblasts and epidermal keratinocytes.

In this study, we used B16-F10 melanoma cells as an experimental model to investigate how the microscale topographic features of the cell adhesion surface are associated with changes in melanoma cell morphology and melanogenic activity. Polydimethylsiloxane (PDMS) substrates patterned with pillar- and hole-shaped microstructures of defined diameters (5, 10, and 50 μm) and heights or depths (10 μm) were fabricated to systematically vary the physical constraints imposed on adherent cells. B16-F10 melanoma cells were cultured on these micropatterned substrates to assess the combined effects of topography and its interplay with the ECM composition. We demonstrated that microscale pillar structures with narrow spacing restricted cell spreading, shortened the distance between cellular protrusions and the nucleus, and induced nuclear shrinkage and deformation. These morphological and nuclear changes were accompanied by a reduction in melanin production and downregulation of melanogenesis-related gene expression, including MITF and key melanogenic enzymes such as tyrosinase (TYR) and Tyr-related proteins (TYRPs) [4,6,7].

Taken together, our findings indicate that microscale topographic cues at the adhesion interface are associated with altered melanoma cell function, highlighting the importance of physical microenvironmental regulation in melanocytic cell biology.

## 2. Materials and Methods

### 2.1. Fabrication of Micropatterned Substrates

The micropatterned cell culture substrates were fabricated by replicating microstructures from a silicon mold on PDMS (Sylgard 184, Dow Corning, Midland, MI, USA). The silicon mold, featuring pillar- and hole-shaped microstructures (diameters and spacings: 5, 10, and 50 μm; height/depth: 10 μm), was purchased from Kyodo International, Inc. (Kawasaki, Japan). The PDMS base and curing agent were mixed at a 10:1 weight ratio, poured onto a silicon mold, and degassed under vacuum. After curing at 60 °C for 2 h, the PDMS membranes were carefully peeled off. For cell adhesion, the substrates were treated with oxygen plasma and coated with either 1 μg/mL of fibronectin (F0895, Sigma-Aldrich, Saint Louis, MO, USA) or Matrigel^®^ Basement Membrane Matrix (356234, Corning, Corning, NY, USA) in PBS at 37 °C for 1 h prior to cell seeding.

### 2.2. Cell Culture

B16-F10 murine melanoma cells were purchased from American Type Culture Collection (ATCC, Manassas, VA, USA) and were cultured in Dulbecco’s Modified Eagle Medium supplemented with 10% fetal bovine serum and 1% penicillin–streptomycin at 37 °C in a humidified atmosphere with 5% CO_2_. The cells were seeded onto micropatterned substrates at a density of 5000 cells/cm^2^. After 24 h, cells were treated with 100 μM of 3-isobutyl-1-methylxanthine (IBMX; 095-03413, Wako Chemicals, Osaka, Japan) for melanogenic stimulation and cultured for the designated periods before analysis.

### 2.3. Observation of Scanning Electron Microscopy

The topography of the PDMS substrates and morphology of the adherent B16-F10 cells were observed using scanning electron microscopy (SEM, SU8600, Hitachi, Tokyo, Japan). The substrates were fixed with 2.5% glutaraldehyde in PBS for 2 h. After washing with distilled water, dehydration was performed using a graded ethanol series, followed by critical-point drying. Before observation, the samples were sputter-coated with a gold-palladium alloy. Observations were performed using an SEM at an accelerating voltage of 5 kV.

### 2.4. Immunofluorescence Staining

For immunofluorescence staining, the cells cultured on the substrates for 24 h were fixed with 4% paraformaldehyde, permeabilized with 0.1% Triton X-100, and blocked with 1% bovine serum albumin. For visualization of focal adhesions, cytoskeleton, and nuclei, samples were incubated with monoclonal anti-vinculin antibody produced in mouse (V4505, Sigma-Aldrich) overnight at 4 °C, followed by Goat anti-Mouse IgG (H + L) Cross-Adsorbed Secondary Antibody, Alexa Fluor™ 555 (A21422, Invitrogen, Carlsbad, CA, USA), Alexa Fluor 488-conjugated phalloidin (A12379, Invitrogen), and Hoechst 33,342 (H342, Dojindo Molecular Technologies, Kumamoto, Japan) for 1 h at room temperature. Fluorescence images were captured using a fluorescence microscope (IX73, Olympus, Tokyo, Japan) with a 40× objective lens (LCACHN40×IPC, Olympus). Quantitative analyses of cell area, protrusion length, and nuclear morphometrics (area, circularity, solidity, and aspect ratio) were performed using ImageJ/Fiji software (https://fiji.sc/, NIH, Bethesda, MD, USA). Circularity (4π × area/perimeter^2^) ranges from 0 to 1, where a value of 1 indicates a perfect circle and lower values reflect increasingly elongated or irregular shapes. Solidity (area/convex area) represents the ratio between the nuclear area and its convex hull area, providing a measure of boundary irregularity. Values close to 1 indicate a smooth outline, whereas lower values indicate indentation or deformation of the nuclear boundary. The nuclear aspect ratio (major axis/minor axis) was quantified as an additional descriptor to distinguish nuclear elongation from boundary irregularity. At least 50 cells from at least three independent experiments per condition were analyzed.

### 2.5. Time-Lapse Phase-Contrast Microscopy

Time-lapse phase-contrast microscopy was performed to analyze the melanoma cell adhesion dynamics and migration on the micropatterned substrates. Melanoma cells were seeded onto fibronectin-coated flat or micropatterned PDMS substrates and allowed to adhere for 1 h under standard culture conditions. Following initial attachment, time-lapse imaging was initiated and continued for 24 h. Phase-contrast images were acquired using an inverted microscope (IX83, Olympus) equipped with a 10× objective lens (CACHN10× IPC, Olympus) with a cell culture system maintained at 37 °C and 5% CO_2_. Images were collected at 20 min intervals. Quantitative analysis of the cell migration distance was performed using ImageJ/Fiji software (NIH). At least 50 cells from at least three independent experiments per condition were analyzed.

### 2.6. Quantification of Melanin Content

The melanin content was determined using a modified NaOH dissolution method. After 72 h of culture, the B16-F10 melanoma cells were harvested from the substrates using 0.25% trypsin-EDTA, and the total cell number was counted. The cells were then lysed in 1 M NaOH containing 10% dimethyl sulfoxide at 90 °C for 1 h to dissolve the melanin. To quantify melanin concentration, a standard calibration curve was prepared using synthetic melanin (M0418, Sigma-Aldrich) dissolved in the same lysis solution at concentrations ranging from 0 to 1 mg/mL. The absorbance of each sample and the standard was measured at 405 nm using a spectrophotometer (V-630Bio, JASCO, Tokyo, Japan). The melanin concentration was normalized to the total cell number. The values from each independent experiment were expressed relative to the flat surface without IBMX (set to 1.0) to account for inter-assay variability.

### 2.7. Quantitative Real-Time PCR

Total RNA was extracted using the TRIzol Reagent (15596026, Invitrogen) according to the manufacturer’s protocol, followed by reverse transcription to yield cDNA using the ReverTra Ace qPCR RT Master Mix (FSQ-201, TOYOBO, Osaka, Japan). Real-time PCR was performed using the SYBR Green PCR Master Mix (QPS-201, TOYOBO) on an Agilent Stratagene Mx3000P QPCR system. The expression levels of melanogenic genes (*Mitf*, *Tyr*, and *Trp1*) were normalized to the housekeeping gene *Gapdh* using the 2^−ΔΔCt^ method. The primer sequences used were as follows: *GAPDH* (housekeeping gene, forward; 5′-AAATGGTGAAGGTCGGTGTG-3′, reverse; 5′-TGAAGGGGTCGTTGATGG-3′), *Mitf* (forward; 5′-CAGAGTCTGAAGCAAGAGCATTG-3′, reverse; 5′-CTTGTTCCACCGCATGTCTG-3′), *Tyr* (forward; 5′-GTCCACTCACAGGGATAGCAG-3′, reverse; 5′-TGGCTTCTGGGTAAACTTCCA-3′) and *Trp1* (forward; 5′-GTGAGCAGCTCTGTGCTGTATT-3′, reverse; 5′TATTGGCACACTCTCGTGGAA-3′).

### 2.8. Statistical Analysis

Data are presented as mean ± standard deviation. For the morphological analyses, at least three independent biological replicates were performed for each condition, and a minimum of 50 cells or nuclei per condition were analyzed. For melanin content and gene analyses, five independent experiments were conducted. Prior to parametric testing, the normality of data distribution for each dataset was assessed using the Shapiro–Wilk test. For datasets satisfying the normality assumption, statistical significance was evaluated using one-way ANOVA followed by Fisher’s least significant difference post hoc test for comparisons among groups. For datasets that did not satisfy the normality assumption, the Kruskal–Wallis test followed by Dunn’s test was applied. Statistical significance was set at a *p*-value less than 0.05. All statistical analyses were performed using GraphPad Prism software (https://www.graphpad.com/, GraphPad Software, San Diego, CA, USA).

## 3. Results

### 3.1. Fabrication of PDMS Micropatterned Substrates and Morphological Responses of Melanoma Cells Observed by SEM

To examine the influence of microscale topographic features at the cell adhesion surface on melanoma cell morphology, PDMS substrates with defined pillar- and hole-shaped microstructures were fabricated. The microstructures were designed with diameters and inter-structural spacings of 5, 10, or 50 μm, and a uniform height or depth of 10 μm (pillar-shaped P5, P10, and P50, and hole-shaped H5, H10, and H50). SEM observations confirmed the high-fidelity reproduction of the intended topographies across all patterns (Figure S1).

**Figure 1:**
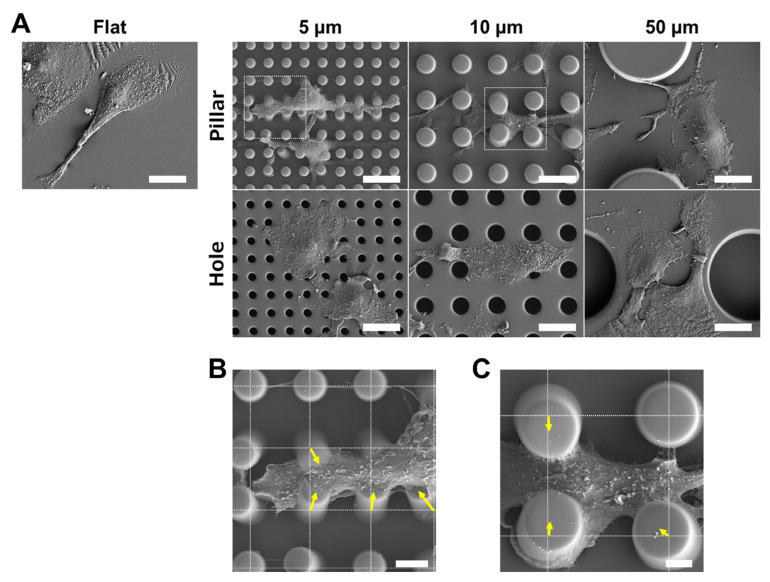
SEM observation of B16-F10 melanoma cell morphology on micropatterned substrates. (**A**) Representative SEM images showing the morphology of cells cultured on flat surfaces and on micropatterned substrates with pillar- or hole-shaped microstructures. Scale bar: 20 µm. (**B**,**C**) Higher-magnification SEM images of cells interacting with pillar structures of (**B**) 5 µm and (**C**) 10 µm diameter, corresponding to the dotted boxs in (**A**). Yellow arrows indicate the direction of cell-generated forces exerted on the pillars at the cell–substrate interface. Scale bar: 5 µm.

B16-F10 cells were cultured for 24 h on fibronectin-coated substrates and subsequently observed using SEM (Figure 1A). On P5 and P10, a distinct bending of the PDMS pillars beneath or adherent to the cells was observed (Figure 1B,C), suggesting the presence of cell-generated mechanical forces at the cell–substrate interface. In contrast, the cells cultured on H5 and H10 exhibited extensive cell spreading, which was largely unaffected by the presence of holes. These observations indicate that melanoma cells respond differently to convex (pillar) and concave (hole) microscale topographies at adhesion interfaces.

### 3.2. Micropattern-Dependent Regulation of Cell Spreading, Protrusion, and Migration

**Figure 2:**
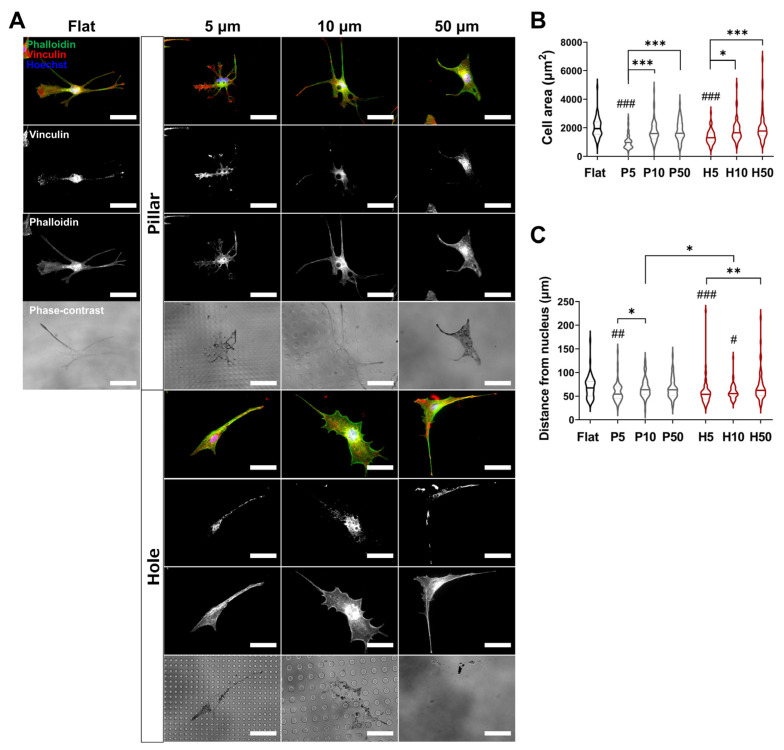
Morphological responses of B16-F10 melanoma cells on micropatterned substrates. (**A**) Representative fluorescence microscopy (**upper**) of actin (red), vinculin (green) and nuclei (blue) and phase-contrast microscopy (**below**) in cells on each substrate. Scale bar: 50 μm. (**B**,**C**) Quantification of (**B**) projected cell area and (**C**) the maximum distance from the nucleus to the distal tip of cellular protrusions on each substrate. *n* > 50 cells per condition. Kruskal–Wallis test, * *p* < 0.05, ** *p* < 0.01, and *** *p* < 0.001 compared with indicated groups; # *p* < 0.05, ## *p* < 0.01, and ### *p* < 0.001 compared with the flat surface. (**B**) Cell area: ### *p* < 0.0001 (Flat vs. P5), ### *p* < 0.0001 (Flat vs. H5), *** *p* < 0.0001 (P5 vs. P10), *** *p* < 0.0001 (P5 vs. P50), * *p* = 0.0388 (H5 vs. H10), and *** *p* = 0.0006 (H5 vs. H50). (**C**) Distance from the nucleus: ## *p* = 0.0057 (Flat vs. P5), ### *p* = 0.0006 (Flat vs. H5), # *p* = 0.0128 (Flat vs. H10), * *p* = 0.0122 (P5 vs. P10), * *p* = 0.0260 (P10 vs. H10), and ** *p* = 0.0062 (H5 vs. H50).

To quantitatively evaluate the effects of microscale topography on cell morphology, we performed immunofluorescence staining for the cytoskeletal and cell-adhesive components (Figure 2A). On P5, B16-F10 cells exhibited reduced spreading relative to flat substrates, extending their protrusions while avoiding direct occupation of the pillar regions. In contrast, the cells on H5 and H10 spread continuously across the surface, consistent with the SEM observations. For H50, some cells were completely accommodated within the holes (Figure S2). In addition, vinculin immunofluorescence revealed an increased proportion of vinculin-positive area on P5 relative to that on flat substrates (Figure S3).

Quantitative image analysis revealed that the cell area was significantly reduced on P5 and H5 compared to the flat controls (Figure 2B), indicating reduced cell spreading on microscale patterns with narrow topographical spacing. In addition, the distance from the nucleus to the distal tips of the cellular protrusions was significantly shortened on P5, H5, and H10 (Figure 2C). These results demonstrate that the microscale topography alters both the extent of cell spreading and the spatial organization of protrusive structures.

**Figure 3:**
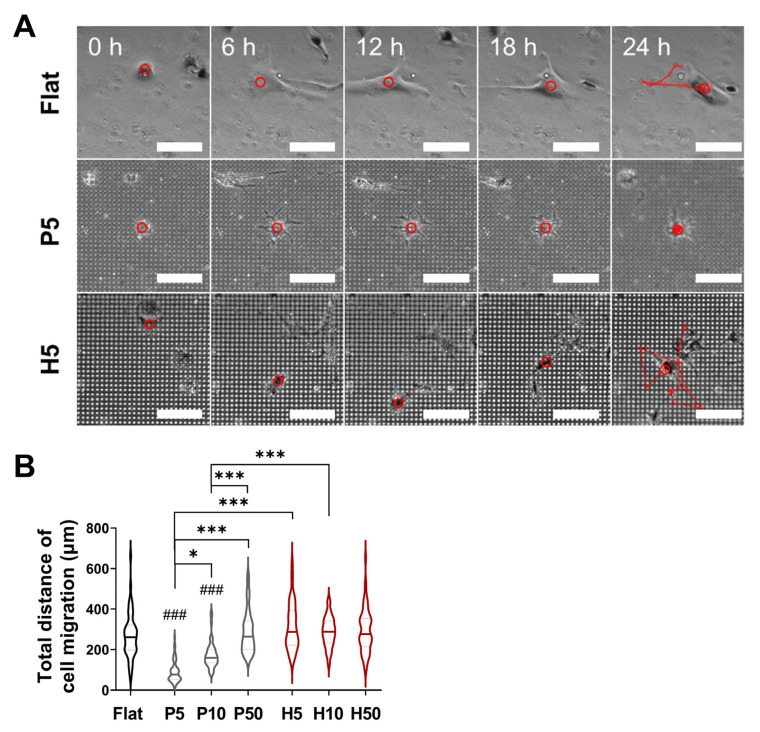
Micropattern-induced reduction in cell motility. (**A**) Time-lapse images of cells migrating on flat (**upper**), P5 (**middle**), and H5 (**below**) substrates. Images are acquired every 20 min for 24 h; cell positions after 0, 6, 12, 18, or 24 h of recording are shown from left to right. Red circles indicate nuclear positions, and red lines in the 24 h images indicate migration trajectories. Scale bar: 100 μm. (**B**) Quantification of the total migration distance of cells on each substrate. *n* > 50 cells per condition. Kruskal–Wallis test, * *p* < 0.05, *** *p* < 0.001 compared with indicated groups; ### *p* < 0.001 compared with the flat surface. ### *p* < 0.0001 (Flat vs. P5), ### *p* < 0.0001 (Flat vs. P10), * *p* = 0.0195 (P5 vs. P10), *** *p* < 0.0001 (P5 vs. P50), *** *p* < 0.0001 (P5 vs. H5), *** *p* < 0.0001 (P10 vs. P50), and *** *p* < 0.0001 (P10 vs. H10).

To further investigate the effects of topography on early adhesion dynamics and cell motility, we performed time-lapse phase-contrast microscopy of cells cultured on flat and micropatterned substrates for 24 h (Figure 3A, Supplementary Movie S1). Cells cultured on P5 and P10 exhibited markedly reduced migration throughout the observation period compared with other substrates (Figure 3B). This reduction in cell motility indicates that the microscale pillar topographies not only restrict static cell spreading but also inhibit dynamic migratory behavior.

### 3.3. Micropattern-Induced Nuclear Deformation and Size Reduction

**Figure 4:**
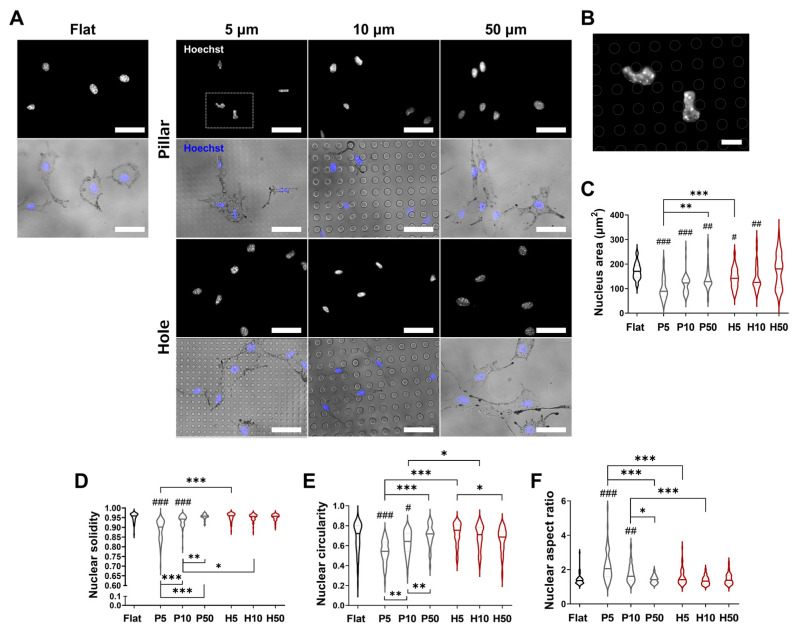
Micropattern-induced deformation in nuclear morphology of B16-F10 melanoma cells. (**A**) Representative fluorescence microscopy images of nuclei (**upper panels**) and corresponding phase-contrast merged images (**lower panels**) of cells cultured on each substrate. Scale bar: 50 µm. (**B**) Higher-magnification image of a deformed nucleus observed on a pillar-patterned substrate with 5 µm diameter pillars, corresponding to the boxed region in (**A**). Scale bar: 10 µm. (**C**–**F**) Quantitative analysis of nuclear (**C**) area, (**D**) solidity, (**E**) circularity, and (**F**) aspect ratio on each substrate. *n* > 50 nuclei per condition. Kruskal–Wallis test, * *p* < 0.05, ** *p* < 0.01, and *** *p* < 0.001 compared with indicated groups; # *p* < 0.05, ## *p* < 0.01, and ### *p* < 0.001 compared with the flat surface. (**C**) Nuclear area: ### *p* < 0.0001 (Flat vs. P5), ### *p* < 0.0001 (Flat vs. P10), ## *p* = 0.0032 (Flat vs. P50), # *p* = 0.0317 (Flat vs. H5), ## *p* = 0.0045 (Flat vs. H10), *** *p* = 0.0020 (P5 vs. P50), and *** *p* < 0.0001 (P5 vs. H5). (**D**) Nuclear solidity: ### *p* < 0.0001 (Flat vs. P5), ### *p* = 0.0008 (Flat vs. P10), *** *p* = 0.0008 (P5 vs. P10), *** *p* < 0.0001 (P5 vs. P50), *** *p* < 0.0001 (P5 vs. H5), ** *p* = 0.0011 (P10 vs. P50), and * *p* = 0.0150 (P10 vs. H10). (**E**) Nuclear circularity: ### *p* < 0.0001 (Flat vs. P5), # *p* = 0.0118 (Flat vs. P10), ** *p* = 0.0019 (P5 vs. P10), *** *p* < 0.0001 (P5 vs. P50), *** *p* < 0.0001 (P5 vs. H5), ** *p* = 0.0042 (P10 vs. P50), * *p* = 0.0103 (P10 vs. H10), and * *p* = 0.0167 (H5 vs. H50). (**F**) Nuclear aspect ratio: ### *p* < 0.0001 (Flat vs. P5), ## *p* = 0.0012 (Flat vs. P10), *** *p* < 0.0001 (P5 vs. P50), *** *p* < 0.0001 (P5 vs. H5), * *p* = 0.0136 (P10 vs. P50), and *** *p* < 0.0001 (P10 vs. H10).

Because the micropillar spacing on the P5 and P10 substrates is comparable to the nuclear dimensions of B16-F10 cells, we hypothesized that these structures may impose direct physical constraints on the nucleus and therefore examined the nuclear morphology in detail (Figure 4A). Marked changes in the nuclear shape were observed on P5 and P10 (Figure 4A,B). The nuclear area was significantly reduced on most substrates (P5, P10, P50, H5, and H10) relative to flat substrates (Figure 4C).

In addition to size reduction, marked changes in the nuclear shape were observed on P5 and P10 (Figure 4A,B). To quantitatively evaluate nuclear deformation, two shape descriptors, circularity and solidity, were analyzed using ImageJ [25,26]. Quantitative analysis showed significant decreases in nuclear circularity and solidity compared to the flat controls, indicating increased nuclear deformation and irregularity (Figure 4D,E). To further characterize the nuclear shape changes, the nuclear aspect ratio was quantified as an additional descriptor of elongation. B16-F10 cells on P5 and P10 exhibited significantly increased nuclear aspect ratio compared to the flat controls (Figure 4F). These changes were less pronounced on the hole-patterned substrates, suggesting that convex microtopographies impose greater biophysical constraints on the nucleus than concave structures.

### 3.4. Topographical Reduction in Melanin Production and Melanogenesis-Related Gene Expression

**Figure 5:**
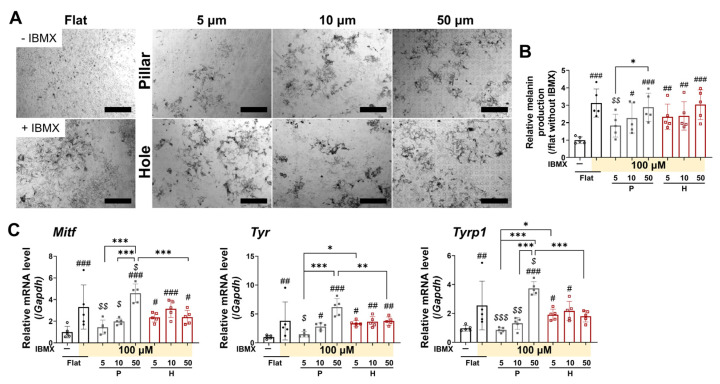
Topographical reduction in melanogenesis in B16-F10 melanoma cells. (**A**) Representative phase-contrast microscopy images of cells cultured for 4 days on flat and micropatterned substrates, showing differences in intracellular melanin accumulation. Scale bar: 500 µm. (**B**) Quantification of melanin content in cells cultured on each substrate. Data are normalized to the flat surface without IMBX. (**C**) Quantitative RT-PCR analysis of melanogenesis-related gene expression, including *Mitf*, *Tyr*, and *Tyrp1*, in cells cultured on each substrate. Gene expression levels are normalized to housekeeping gene (*Gapdh*) and expressed relative to the flat surface without IBMX. *n* = 5. One-way ANOVA test, * *p* < 0.05, ** *p* < 0.01, and *** *p* < 0.001 compared with indicated groups; # *p* < 0.05, ## *p* < 0.01, and ### *p* < 0.001 compared with the flat surface without IBMX; *$ p* < 0.05, *$$ p* < 0.01, and *$$$ p* < 0.001 compared with the flat surface with IBMX. (**B**) Quantification of melanin content: ### *p* < 0.0001 (Flat without IBMX vs. Flat with IBMX), # *p* = 0.0117 (Flat without IBMX vs. P10), ### *p* = 0.0004 (Flat without IBMX vs. P50), ## *p* = 0.0080 (Flat without IBMX vs. H5), ## *p* = 0.0061 (Flat without IBMX vs. H10), ### *p* = 0.0001 (Flat without IBMX vs. H50), *$$ p* = 0.0099 (Flat with IBMX vs. P5), and * *p* = 0.0331 (P5 vs. P50). (**C**) *Mitf* expression: ### *p* = 0.0004 (Flat without IBMX vs. Flat with IBMX), ### *p* < 0.0001 (Flat without IBMX vs. P50), # *p* = 0.0277 (Flat without IBMX vs. H5), ### *p* = 0.0009 (Flat without IBMX vs. H10), # *p* = 0.0255 (Flat without IBMX vs. H50), *$$ p* = 0.0032 (Flat with IBMX vs. P5), *$ p* = 0.0286 (Flat with IBMX vs. P10), *$ p* = 0.0381 (Flat with IBMX vs. P50), *** *p* < 0.0001 (P5 vs. P50), *** *p* < 0.0001 (P10 vs. P50), *** *p* = 0.0007 (P50 vs. H50), *Tyr* expression: ## *p* = 0.0030 (Flat without IBMX vs. Flat with IBMX), # *p* = 0.0437 (Flat without IBMX vs. P10), ### *p* < 0.0001 (Flat without IBMX vs. P50), # *p* = 0.0102 (Flat without IBMX vs. H5), ## *p* = 0.0047 (Flat without IBMX vs. H10), ## *p* = 0.0032 (Flat without IBMX vs. H50), *$ p* = 0.0122 (Flat with IBMX vs. P5), *** *p* < 0.0001 (P5 vs. P50), * *p* = 0.0372 (P5 vs. H5), ** *p* = 0.0099 (P50 vs. H50), *Tyrp1* expression: ## *p* = 0.0014 (Flat without IBMX vs. Flat with IBMX), ### *p* < 0.0001 (Flat without IBMX vs. P50), # *p* = 0.0447 (Flat without IBMX vs. H5), # *p* = 0.0115 (Flat without IBMX vs. H10), *$$$ p* = 0.0007 (Flat with IBMX vs. P5), *$$ p* = 0.0100 (Flat with IBMX vs. P10), *$ p* = 0.0125 (Flat with IBMX vs. P50), *** *p* < 0.0001 (P5 vs. P50), * *p* = 0.0260 (P5 vs. H5), *** *p* < 0.0001 (P10 vs. P50), and *** *p* < 0.0001 (P50 vs. H50).

To assess whether the micropattern-induced changes in cell spreading and nuclear morphology were associated with functional alterations in melanogenesis, melanin production and melanogenesis-related gene expression were evaluated (Figure 5A). Cells were stimulated with IBMX, a cAMP phosphodiesterase inhibitor known to enhance melanin synthesis [27], and the melanin content was quantified. Cells cultured on P5 exhibited a significant reduction in melanin production compared with IBMX-treated cells on flat substrates (Figure 5B). Although cells on P10, H5, and H10 showed a decreasing trend in melanin content, these differences were not statistically significant.

RT-PCR analysis further showed that the expression of key melanogenic genes, including *Mitf*, *Tyr*, and *Tyrp1*, was significantly downregulated on P5 compared to that in IBMX-treated cells on flat substrates (Figure 5C). On the P10 substrates, *Mitf* and *Tyrp1* expression levels were also significantly reduced. In contrast, on P50 substrates, the expression levels of *Mitf*, *Tyr*, and *Tyrp1* were significantly increased relative to those in the flat IBMX-treated group. No significant differences in the expression of these genes were observed in the hole-shaped patterns compared with the IBMX-treated flat control. Taken together, these results indicate that narrow micropillars affect the transcriptional program underlying melanogenesis in a topography-dependent manner, with the strongest effects observed on P5 substrates.

### 3.5. Effects of ECM Composition on Micropattern-Induced Regulation of Melanoma Cell Morphology and Function

**Figure 6:**
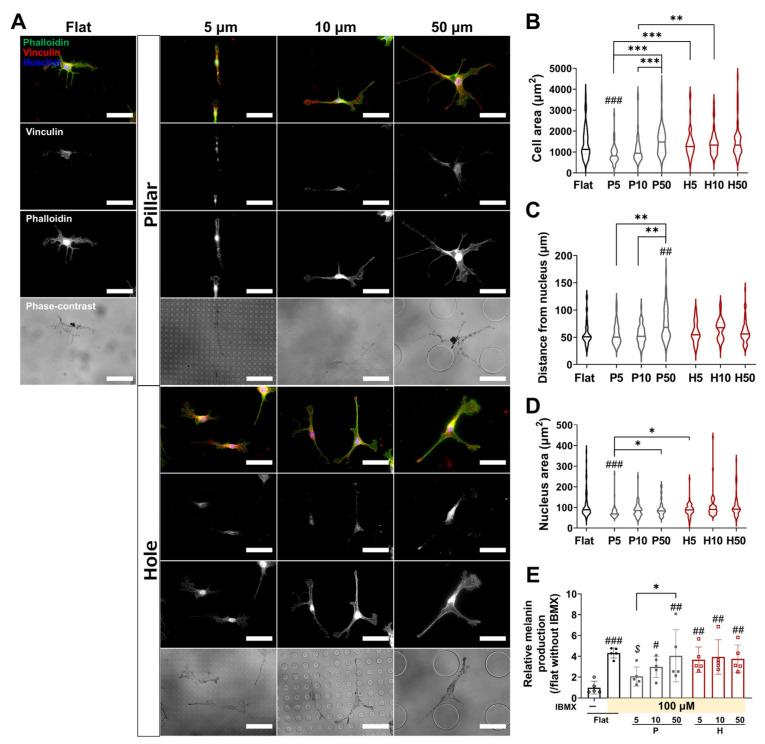
Effects of ECM composition on micropattern-induced regulation of B16-F10 melanoma cell morphology and function. (**A**) Representative fluorescence microscopy (**upper**) of actin (red), vinculin (green) and nuclei (blue) and phase-contrast microscopy (**below**) in cells cultured on Matrigel-coated substrates. Scale bar: 50 µm. (**B**–**E**) Quantitative analysis of (**B**) projected cell area, (**C**) maximum distance from the nucleus to distal cellular protrusions, and (**D**) nuclear area in cells cultured on Matrigel-coated substrates. (**B**–**D**) *n* > 50 cells or nuclei per condition. Kruskal–Wallis test, * *p* < 0.05, ** *p* < 0.01, and *** *p* < 0.001 compared with indicated groups; ## *p* < 0.01, ### *p* < 0.001 compared with the flat surface. (**B**) Cell area: ### *p* < =0.0003 (Flat vs. P5), *** *p* < 0.0001 (P5 vs. P50), *** *p* = 0.0001 (P5 vs. H5), *** *p* = 0.0004 (P10 vs. P50), and ** *p* = 0.0089 (P10 vs. H10). (**C**) Distance from the nucleus: ## *p* = 0.0061 (Flat vs. P50), ** *p* = 0.0042 (P5 vs. P50), and ** *p* = 0.0036 (P10 vs. P50). (**D**) Nuclear area: ### *p* < 0.0001 (Flat vs. P5), * *p* = 0.0485 (P5 vs. P50), and * *p* = 0.0117 (P5 vs. H5). (**E**) Quantification of melanin content in cells cultured on Matrigel-coated substrates. Data are normalized to the flat surface without IMBX. *n* = 5. One-way ANOVA test, * *p* < 0.05 compared with indicated groups; ## *p* < 0.01, ### *p* < 0.001 compared with the flat surface without IBMX; *$ p* < 0.05 compared with the flat surface with IBMX. ### *p* = 0.0005 (Flat without IBMX vs. Flat with IBMX), # *p* = 0.0275 (Flat without IBMX vs. P10), ## *p* = 0.0012 (Flat without IBMX vs. P50), ## *p* = 0.0040 (Flat without IBMX vs. H5), ## *p* = 0.0017 (Flat without IBMX vs. H10), ## *p* = 0.0030 (Flat without IBMX vs. H50), *$ p* = 0.0132 (Flat with IBMX vs. P5), and * *p* = 0.0278 (P5 vs. P50).

To examine whether ECM composition modulates the effects of microscale topography, B16-F10 cells were cultured on micropatterned substrates coated with Matrigel basement membrane matrix. Immunofluorescence analysis showed that, similar to fibronectin-coated conditions, the cell area was significantly reduced on P5compared to that on flat substrates (Figure 6A–C). The nuclear area was significantly decreased on P5 relative to the flat controls (Figure 6D), and nuclear solidity was decreased on P5 (Figure S4). Melanin production was also significantly reduced on P5 under Matrigel-coated conditions (Figure 6E), and *Mitf* gene expression was decreased (Figure S5). Together, these results suggest that, while ECM composition can influence specific aspects of cell morphology [11], microscale pillar topography exerts consistent topography-associated effects on melanoma cell melanogenic function across ECM conditions.

## 4. Discussion

In this study, we demonstrated that microscale topographic features of the cell adhesion interface are associated with altered B16-F10 melanoma cell morphology, nuclear architecture, and melanogenic activity. Using PDMS substrates with defined pillar- and hole-shaped microstructures, we show that 5-μm pillar patterns (diameter and spacing) impose significant physical constraints on melanoma cells, coinciding with restricted cell spreading, nuclear deformation, and reduction in melanin production. These findings highlight the role of adhesion surface topography in the mechanoregulation of melanoma cell function.

Morphological analyses revealed that micropillar substrates with small feature sizes markedly restricted cell spreading and altered the protrusive architecture, whereas hole-shaped patterns of comparable dimensions exerted weaker effects. SEM and immunofluorescence imaging consistently showed that cells tightly packed the space between the closely spaced pillars, resulting in reduced cell area and altered cytoskeletal organization. This behavior is consistent with previous reports demonstrating that cells sense and respond to geometric confinement imposed by microtopographical surfaces, thereby adapting their overall morphology across diverse cell types [15,17,28,29,30]. For instance, the differentiation of human epidermal keratinocytes is stimulated on micropillar-patterned substrates, which is accompanied by a decrease in the nuclear localization of the mechanosensitive transcriptional regulator YAP1 compared to flat substrates [30]. In contrast, our results in B16-F10 melanoma cells showed reduced melanogenic activity and downregulation of melanogenesis-related genes under micropillar confinement. These apparently distinct responses likely reflect cell type-dependent differences in mechanobiological regulation, whereby identical topographic cues induce different functional phenotypes depending on cellular context. In the context of melanoma biology, the reduced expression of melanogenesis-related genes may also reflect altered melanoma cell plasticity or a shift toward a less differentiated melanoma cell state [4,5]. Although melanoma lineage plasticity was not directly examined in the present study, our findings suggest that microscale topographic cues at the adhesion interface may contribute to the regulation of melanoma cell phenotypic state through mechanobiological pathways.

In addition to static morphological changes, we observed a pronounced reduction in cell migration on P5 and P10, as revealed by 24 h time-lapse phase-contrast microscopy. Cells cultured on narrow pillar arrays exhibited limited displacement from their initial attachment sites and reduced motility compared with cells on flat or hole-patterned substrates. This finding suggests that microscale pillar topographies not only constrain cell spreading but also impair effective migration. Similar topography-dependent suppression of cell motility has been reported in various cell types and is often attributed to the excessive stabilization of focal adhesions and reduced protrusion dynamics under spatial confinement [15,17,31,32]. Consistent with this interpretation, quantification of the vinculin-positive area revealed an increased vinculin signal fraction in B16-F10 cells on P5 relative to flat substrates (Figure S3), suggesting an altered adhesion-related protein distribution under topographic confinement. However, vinculin localization alone is insufficient to define focal adhesion status, as increased vinculin-positive areas may reflect changes in protein expression level, cytoplasmic redistribution, or differences in the cell spreading area rather than an increase in discrete focal adhesion structures [33,34]. Co-staining with established focal adhesion markers, such as phosphorylated focal adhesion kinase, together with quantitative analysis of adhesion number, size, and turnover dynamics, will therefore be required in future studies to clarify the underlying mechanisms.

Notably, the different responses to pillar- and hole-shaped patterns suggest that convex topographies impose stronger mechanical constraints than concave topographies. The migration analysis shown in Figure 3 indicates that the P5 and P10 substrates significantly constrained cell migration, whereas H5 slightly increased the migration distance, and H10 showed no significant difference compared with the flat surface. Similar asymmetrical responses to convex and concave microstructures have been reported in other cell types and are thought to arise from differences in adhesion continuity and the spatial distribution of traction forces at the cell–substrate interface [31,35].

We also observed pronounced nuclear shrinkage and deformation in cells cultured on micropillar substrates with narrow spacing. Given that the dimensions of these microstructures are comparable to the nuclear size, the observed reductions in the nuclear area, circularity, and solidity suggest that the physical confinement at the adhesion interface is mechanically transmitted to the nucleus. This interpretation is consistent with the concept of mechanical coupling between the cytoskeleton and nucleus via the linker of nucleoskeleton and cytoskeleton complex, through which extracellular mechanical cues can influence nuclear shape and mechanics [36,37].

Previous studies have shown that mechanical deformation of the nucleus can influence gene regulation by altering chromatin organization and transport of regulatory molecules between the nucleus and cytoplasm [36,38]. Nuclear deformation can also affect the localization of mechanosensitive transcriptional regulators such as YAP/TAZ, which respond to mechanical cues in the cellular environment [39,40,41]. In melanocytic cells, the nuclear accumulation of YAP has been reported to increase in a stiffness-dependent manner [11,14]. Although YAP/TAZ activity was not directly examined in this study, our results showed that the micropillar substrates induced pronounced nuclear deformation and size reduction, particularly on P5 and P10, along with decreased melanogenic gene expression. These findings suggest that microscale topographic constraints may influence melanoma cell function by mechanically altering the nuclear structure, which in turn could affect the activity or localization of mechanosensitive transcriptional regulators and downstream melanogenic gene expression. While our data revealed correlations between surface topography, nuclear deformation, and the reduction in melanogenic activity, the underlying mechanistic pathways were not directly validated in the present study. Future studies investigating YAP/TAZ localization, chromatin organization, and cytoskeletal tension are required to establish the causal relationships.

The present study had several limitations. First, B16-F10 cells are a murine melanoma-derived cell line, and their responses may not fully reflect those of normal primary melanocytes. Therefore, validation using primary human melanocytes is necessary to determine the physiological relevance. Secondly, the current in vitro experiment did not recapitulate the key features of the in vivo pigmentation unit, including melanosome transfer and paracrine interactions between melanocytes and keratinocytes. More physiologically relevant models, such as co-culture systems or three-dimensional skin equivalents, are necessary to address these aspects. Third, the ECM conditions and surface topography employed in this study represent simplified environments and do not fully reproduce the biochemical composition, mechanical properties, or structural complexity of native basement membranes. These limitations restrict the direct translation of our findings to in vivo conditions.

In summary, our findings demonstrate that microscale topographic features of the adhesion surface are associated with altered B16-F10 melanoma cell function, coinciding with constrained cell morphology, nuclear deformation, and melanogenesis reduction. Alternative explanations, such as altered proliferation, metabolic adaptation, or stress responses induced by geometric confinement, cannot be fully excluded and should be addressed in future studies. While these findings are correlative and require mechanistic and translational validation, this study provides a mechanobiological framework for understanding how the physical properties of the microenvironment are associated with melanogenic control and suggests that surface topography is a promising parameter for modulating melanocytic cell behavior in dermatological research and tissue engineering applications.

## Data Availability

The original contributions presented in the study are included in the article/Supplementary Materials, further inquiries can be directed to the corresponding authors.

## Supplementary Materials

The following supporting information can be downloaded at: https://www.mdpi.com/article/10.3390/jfb17060269/s1, Figure S1: Fabrication and structural characterization of micropatterned PDMS substrates. (**A**) Schematic illustrations of micropatterned substrates featuring pillar- and hole-shaped microstructures shown in top and side views. (**B**,**C**) Scanning electron microscopy (SEM) images of the fabricated PDMS substrates with pillar- and hole-shaped microstructures. (**B**) Top-view SEM image. Scale bar: 50 µm. (**C**) SEM image acquired at a 45° tilt angle. Scale bar: 20 µm; Figure S2: B16-F10 melanoma cells accommodated within a hole with 50 μm of diameter. (A) Representative fluorescence microscopy (left) of actin (red), vinculin (green) and nuclei (blue) and phase-contrast microscopy (right) in cells. Scale bar: 50 μm; Figure S3: Vinculin-positive area fraction of B16-F10 melanoma cells on flat and P5 substrates. For measurement, vinculin-positive area fraction was quantified from immunofluorescence images using ImageJ (NIH). First, individual cells were outlined based on actin staining to define the total cell area. Nuclear regions were identified using Hoechst 33342 staining and segmented by automatic thresholding. To exclude perinuclear area, a perinuclear mask was generated by expanding the nuclear boundary by a fixed distance. The resulting region was subtracted from the total cell area to define the peripheral cytoplasmic region for analysis. Vinculin-positive regions were then identified by applying a consistent intensity threshold across all images. The vinculin-positive area over (>0.1 μm^2^) was measured using the “Analyze Particles” function. Over 40 cells per condition were analyzed across three independent experiments. Mann Whitney test, ****p* < 0.001 compared with the flat surface; Figure S4: Nuclear shape parameters of B16-F10 melanoma cells cultured on Matrigel-coated micropatterned substrates. Quantitative analysis of nuclear solidity, circularity, and aspect ratio in cells cultured on Matrigel-coated flat and micropatterned substrates. *n* > 50 nuclei per condition. Kruskal–Wallis test, **p* < 0.05, ***p* < 0.01, ****p* < 0.001 compared with indicated groups; ##*p* < 0.01, ###*p* < 0.001 compared with the flat surface; Figure S5: Expression of melanogenesis-related genes in melanocytes cultured on Matrigel-coated micropatterned substrates. Quantitative RT-PCR analysis of Mitf, Tyr, and Tyrp1 expression in B16-F10 melanoma cells cultured on Matrigel-coated substrates. Gene expression levels were normalized to housekeeping gene (Gapdh) and expressed relative to the flat surface without IBMX. *n* = 5. One-way ANOVA test, #*p* < 0.05, ##*p* < 0.01, ###*p* < 0.001 compared with the flat surface without IBMX; $*p* < 0.05, $$*p* < 0.01 compared with the flat surface with IBMX; **p* < 0.05 compared with indicated groups; Video S1: Migration of melanocytes on flat (upper), P5 (middle), H5 (below) and substrates. All movies are time-lapse phase microscopy images that were taken every 20 min for 24 h. Each movie was generated at 10 fps from one typical area of a time-lapse image sequence.
