# Supplementary material for: Micropillar Topography Regulates Morphology and Melanogenesis in Melanoma Cells
Source: J Funct Biomater. 2026 Jun 1;17(6):269. doi: 10.3390/jfb17060269 (PMC13301322; doi:10.3390/jfb17060269)
Supplement: Supplementary file 1 [file jfb-17-00269-s001.zip › Supplementary materials_melanocyte rev1.docx]

**Supplementary materials**

**
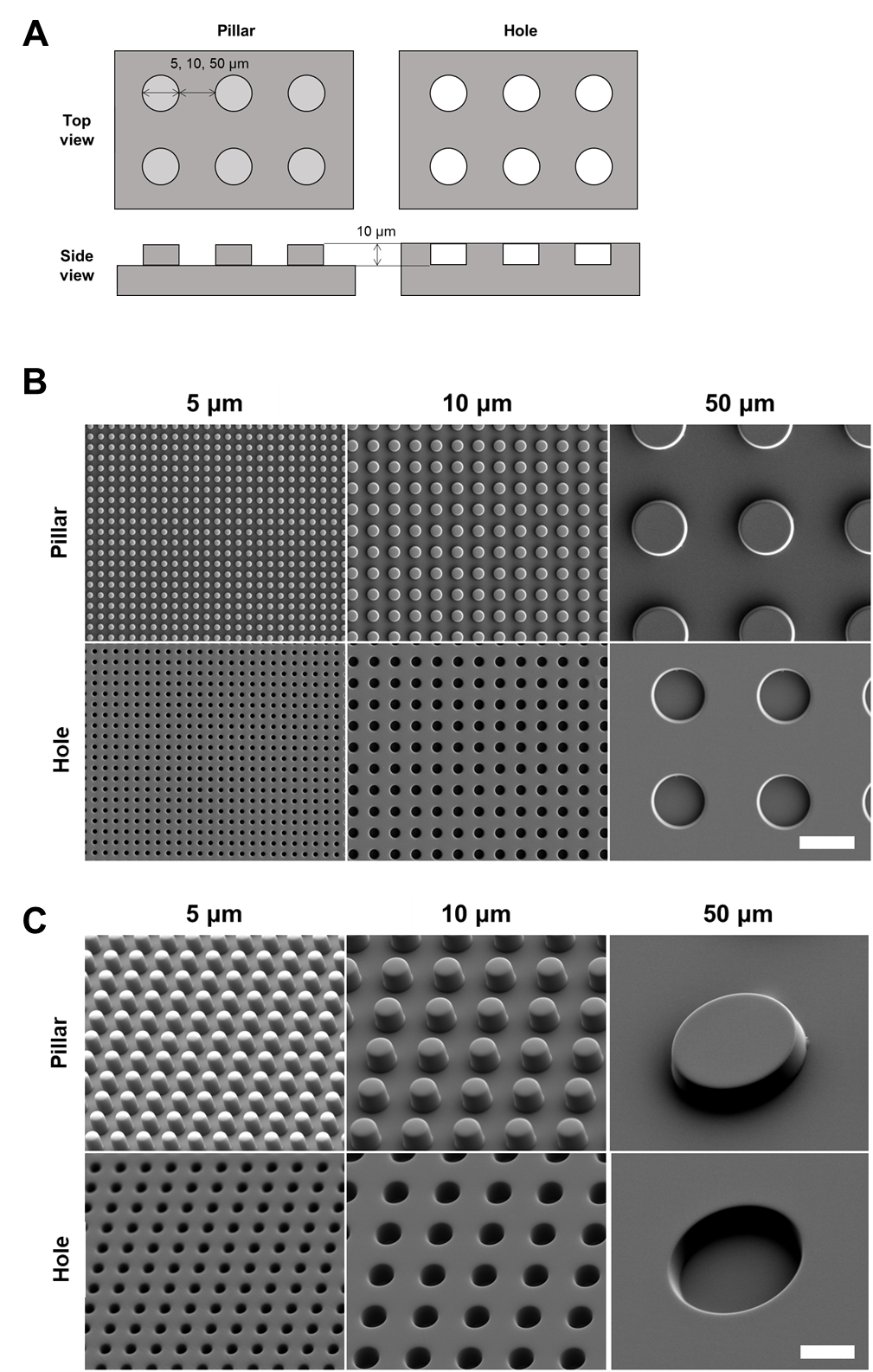
**

Fig. S1. Fabrication and structural characterization of micropatterned PDMS substrates. (A) Schematic illustrations of micropatterned substrates featuring pillar- and hole-shaped microstructures shown in top and side views. (B, C) Scanning electron microscopy (SEM) images of the fabricated PDMS substrates with pillar- and hole-shaped microstructures. (B) Top-view SEM image. Scale bar: 50 µm. (C) SEM image acquired at a 45° tilt angle. Scale bar: 20 µm.


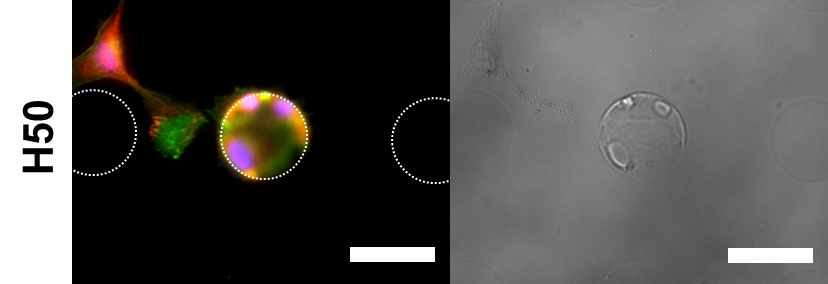


Fig. S2. B16-F10 melanoma cells accommodated within a hole with 50 μm of diameter. (A) Representative fluorescence microscopy (left) of actin (red), vinculin (green) and nuclei (blue) and phase-contrast microscopy (right) in cells. Scale bar: 50 μm.

Fig. S3. Vinculin-positive area fraction of B16-F10 melanoma cells on flat and P5 substrates. For measurement, vinculin-positive area fraction was quantified from immunofluorescence images using ImageJ (NIH). First, individual cells were outlined based on actin staining to define the total cell area. Nuclear regions were identified using Hoechst 33342 staining and segmented by automatic thresholding. To exclude perinuclear area, a perinuclear mask was generated by expanding the nuclear boundary by a fixed distance. The resulting region was subtracted from the total cell area to define the peripheral cytoplasmic region for analysis. Vinculin-positive regions were then identified by applying a consistent intensity threshold across all images. The vinculin-positive area over (>0.1 μm²) was measured using the “Analyze Particles” function. Over 40 cells per condition were analyzed across three independent experiments. Mann Whitney test, ***p < 0.001 compared with the flat surface.


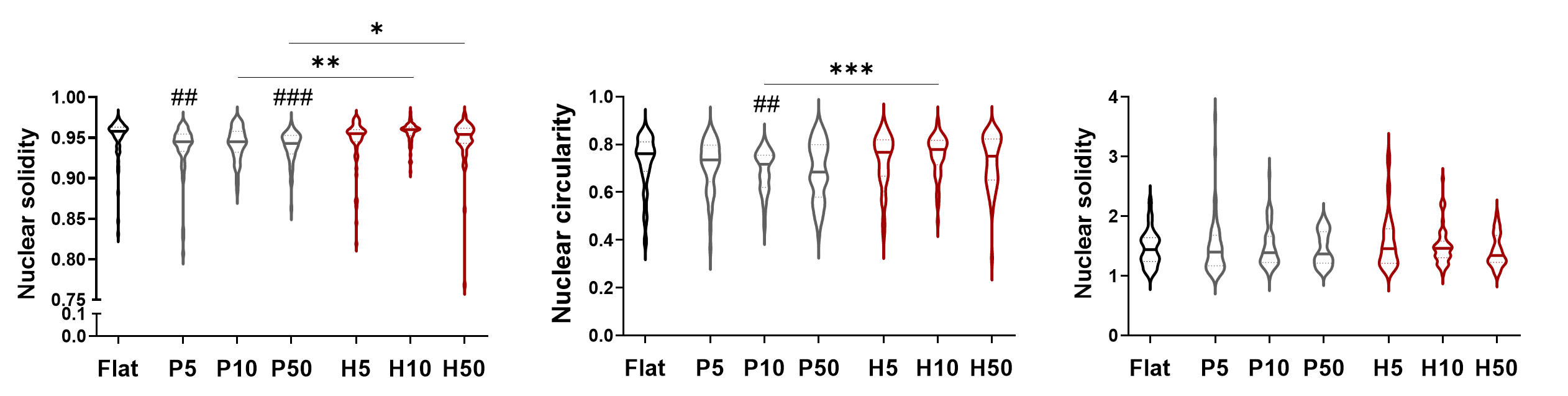


Fig. S4. Nuclear shape parameters of B16-F10 melanoma cells cultured on Matrigel-coated micropatterned substrates. Quantitative analysis of nuclear solidity, circularity, and aspect ratio in cells cultured on Matrigel-coated flat and micropatterned substrates. n > 50 nuclei per condition. Kruskal–Wallis test, *p < 0.05, **p < 0.01, ***p < 0.001 compared with indicated groups; ##p < 0.01, ###p < 0.001 compared with the flat surface.


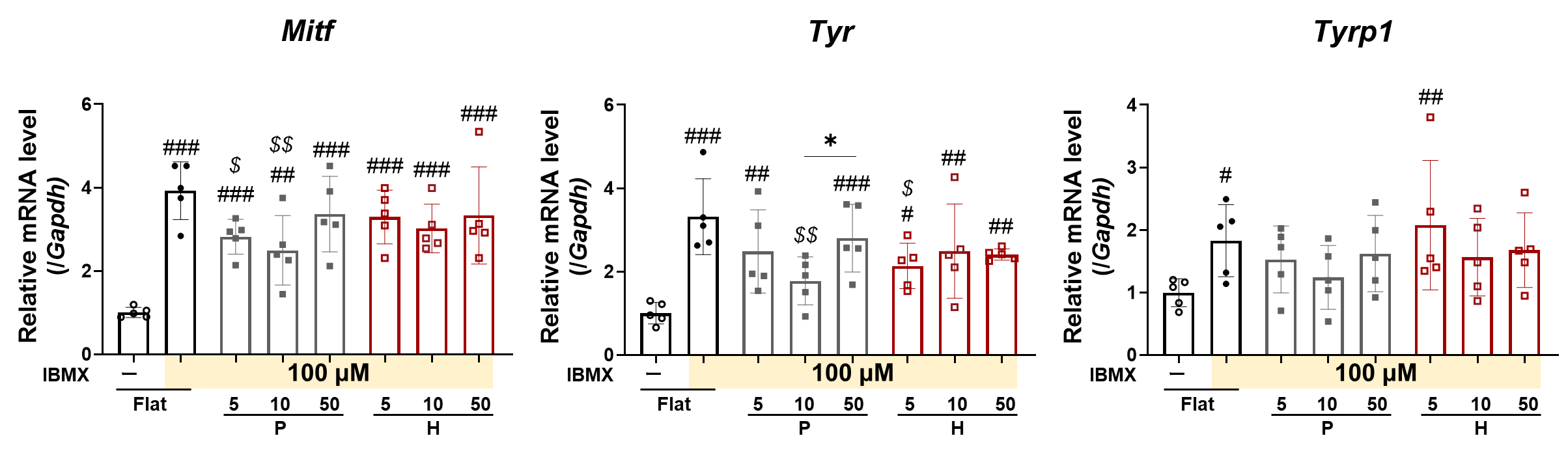


Fig. S5. Expression of melanogenesis-related genes in melanocytes cultured on Matrigel-coated micropatterned substrates. Quantitative RT-PCR analysis of *Mitf*, *Tyr*, and *Tyrp1* expression in B16-F10 melanoma cells cultured on Matrigel-coated substrates. Gene expression levels were normalized to housekeeping gene (*Gapdh*) and expressed relative to the flat surface without IBMX. n = 5. One-way ANOVA test, #p < 0.05, ##p < 0.01, ###p < 0.001 compared with the flat surface without IBMX; *$*p < 0.05, *$$*p < 0.01 compared with the flat surface with IBMX; *p < 0.05 compared with indicated groups.

Supplementary Video S1. Migration of melanocytes on flat (upper), P5 (middle), H5 (below) and substrates. All movies are time-lapse phase microscopy images that were taken every 20 min for 24 h. Each movie was generated at 10 fps from one typical area of a time-lapse

image sequence.
